# Stromal Palladin Expression Is an Independent Prognostic Factor in Pancreatic Ductal Adenocarcinoma

**DOI:** 10.1371/journal.pone.0152523

**Published:** 2016-03-29

**Authors:** Daisuke Sato, Takahiro Tsuchikawa, Tomoko Mitsuhashi, Yutaka Hatanaka, Katsuji Marukawa, Asami Morooka, Toru Nakamura, Toshiaki Shichinohe, Yoshihiro Matsuno, Satoshi Hirano

**Affiliations:** 1 Department of Gastroenterological Surgery II, Hokkaido University Graduate School of Medicine, Sapporo, Japan; 2 Surgical Pathology, Hokkaido University Hospital, Sapporo, Japan; 3 Research Division of Companion Diagnostics, Hokkaido University Hospital, Sapporo, Japan; Fox Chase Cancer Center, UNITED STATES

## Abstract

It has been clear that cancer-associated fibroblasts (CAFs) in the tumor microenvironment play an important role in pancreatic ductal adenocarcinoma (PDAC) progression. However, how CAFs relate to the patients’ prognosis and the effects of chemoradiation therapy (CRT) has not been fully investigated. Tissue microarrays (TMAs) representing 167 resected PDACs without preoperative treatment were used for immunohistochemical studies (IHC) of palladin, α-smooth muscle actin (SMA), and podoplanin. Correlations between the expression levels of these markers and clinicopathological findings were analyzed statistically. Whole sections of surgical specimens from PDACs with and without preoperative CRT, designated as the chemotherapy-first group (CF, n = 19) and the surgery-first group (SF, n = 21), respectively, were also analyzed by IHC. In TMAs, the disease-specific survival rate (DSS) at 5 years for all 167 cases was 23.1%. Seventy cases (41.9%) were positive for palladin and had significantly lower DSS (p = 0.0430). α-SMA and podoplanin were positive in 167 cases (100%) and 131 cases (78.4%), respectively, and they were not significantly associated with DSS. On multivariable analysis, palladin expression was an independent poor prognostic factor (p = 0.0243, risk ratio 1.60). In the whole section study, palladin positivity was significantly lower (p = 0.0037) in the CF group (5/19) with a significantly better DSS (p = 0.0144) than in the SF group (16/22), suggesting that stromal palladin expression is a surrogate indicator of the treatment effect after chemoradiation therapy.

## Introduction

Pancreatic ductal adenocarcinoma (PDAC) is a primary tumor originating from pancreatic duct epithelium and has one of the poorest prognoses of all digestive malignant diseases [1, 2]. The therapeutic standard for PDAC has been surgical resection, but the resection rate is only around 40%. Despite significant improvements in surgery and chemoradiation therapy (CRT) (including adjuvant chemotherapy), the prognosis of patients with PDAC has not changed significantly [3]. Given this background, neoadjuvant CRT and adjuvant surgery for initially unresectable disease are attracting increasing attention as alternatives for the surgery-first method, and reports of their clinical efficacies are increasing [4, 5]. However, in terms of histopathological grading of the treatment effect, many grading systems are not always correlated with patient survival, partly because of difficulty in distinguishing between baseline dense fibrous stroma in PDAC and treatment-induced fibrosis [6, 7].

Recently, fibrous stroma associated with cancer is being increasingly recognized as essential for tumorigenesis in the tumor micro environment. As one of the key players, cancer-associated fibroblasts (CAFs) are activated through interaction with cancer cells, and they express various molecular markers. Their expression is thought to contribute to tumor proliferation, invasion, and migration [8, 9]. Furthermore, CAF marker expression is reported to be correlated with patient prognoses in some epithelial malignancies, [10–12]. Although the most widely accepted marker is α-smooth muscle actin (SMA), there are various other molecular markers. Among them, the actin binding protein palladin is known as a relatively new CAF marker and that has been proven to contribute to CAF differentiation and patient prognosis [13, 14]. Podoplanin, which is recognized as a lymphatic endothelial marker, is reported to be expressed in CAFs of some epithelial malignancies [15]. However, to the best of our knowledge, the association between palladin expression and patients’ prognosis with PDAC have not been previously examined in detail. Furthermore, there has been little study of how CAF markers including palladin and podoplanin are affected by CRT.

The aim of this study was to investigate the clinical implications of CAFs and their modifications after CRT. Surgically resected specimens of patients not treated before surgery and those given CRT before surgery were compared histopathologically.

## Materials and Methods

This study was approved by the Institutional Review Board at Hokkaido University Hospital. All samples were coded to avoid the possibility of patient identification. For all patients, written, informed consent to use the samples for research purposes was obtained.

### Samples from patients

Patients with PDAC who had undergone surgical resection in the Department of Gastroenterological Surgery II at Hokkaido University Hospital between 2000 and 2013 were retrospectively identified via medical records and pathology reports. A total of 167 specimens obtained from patients untreated before surgery were examined using the tissue microarray (TMA) method. The median follow-up period was 19 months (range 2–148 months). On the other hand, 40 specimens obtained from 21 untreated cases before surgery [surgery-first (SF) group] and 19 treated before surgery [chemotherapy-first (CF) group] were also studied using whole sections to observe the possible heterogeneous effects of CRT and CAF marker expression in detail. Selected hematoxylin and eosin-stained (H&E) slides were reviewed by an experienced pathologist (TM) to confirm the original pathological diagnoses and to choose representative areas.

All tumors were staged according to the 7th TMN classification system of the Union for International Cancer Control [16]. The TNM classification and other clinicopathological findings were investigated, and those of TMA cases are shown in S1 Table, while those of the SF and the CF groups are shown in S2 Table. In the CF group, histopathologic treatment effects were assessed both by the Evans grading and the College of American Pathologists (CAP) grading [17, 18]. Grade I—IIa in the Evans grading and grade 2–3 in the CAP grading were regarded as low treatment effect, while grade IIb-IV in the Evans grading and grade 0–1 in the CAP grading were regarded as high treatment effect.

### Tissue microarray analysis

TMA blocks were constructed using a manual tissue microarrayer (JF-4; Sakura Finetek Japan, Tokyo, Japan) with a 2.0-mm-diameter needle from representative areas (both PDAC and pancreatic parenchyma outside the PDAC). The finalized array blocks were sliced into 5-μm-thick serial sections and mounted on glass slides.

### Immunohistochemical evaluation

Tissue sections were deparaffinized in xylene and rehydrated through a graded ethanol series. Heat-induced antigen retrieval was carried out in high-pH antigen retrieval buffer (Dako Cytomation, Glostrup, Denmark). Endogenous peroxidase was blocked by incubation in 3% H_2_O_2_ for 5 min. The primary antibodies against palladin (1:100, 1E6; Novus Biologicals, Colorado, CA, USA), α-smooth muscle actin (SMA) (1:200, 1A4; Abcam, Cambridge, UK), and podoplanin (ready to use, D2-40; Dako), were applied for 60 minutes. These sections were visualized by the HRP-labeled polymer method (EnVision FLEX system, Dako). Immunostained sections were counterstained with hematoxylin, dehydrated in ethanol, and cleared in xylene.

The proportions of stained stromal cells were analyzed. In the TMA study, each core was evaluated to select the area with the highest proportion. In the whole section study, three representative ×200-magnification fields were analyzed, and then the mean proportion was evaluated. Each marker was defined as positive when more than 30% of stromal cells were stained. Two researchers (DS and TM), who were blinded to the patients’ clinical information, independently examined and scored each case. The cut-off value for the positive expression level of palladin was initially set at every 10% from 0% to 100%, and then the log-rank test was used to compare patients with positive and negative palladin expressions. Finally, a cut-off value of 30% was calculated to best reflect patients’ prognosis. The cut-off values of podoplanin and α-SMA were examined in the same way, but they both did not seem to reflect patients’ prognosis, though their cut-off values were determined to be 30%, as for palladin.

### Statistical analysis

Correlations between IHC evaluation and other clinicopathological findings including patient survival were investigated. Disease-specific survival (DSS) and disease-free survival (DFS) were calculated from the date of surgery to the date of the last follow-up or patient death and recurrence. In comparing survival times between the SF group and the CF group, survival time was calculated from the date of initial treatment. Fisher’s exact test and the Wilcoxon test were used to determine dependencies between two variables, as appropriate. Survival curves were estimated by the Kaplan-Meier method and compared using the log-rank test. In DFS analysis of TMA cases, two cases were excluded because of lack of information about the recurrence date. Multivariable analysis was conducted with the factors that were significant on univariate analysis using the Cox proportional hazards regression model. The level for significance was *p* <0.05, and the confidence interval was determined at the 95% level. Statistical analysis was performed using the JMP 11.0 software package (SAS Institute, Inc., Cary, NC, USA) for Windows.

## Results

### CAF marker expression in PDAC with no preoperative therapy

**Fig 1:**
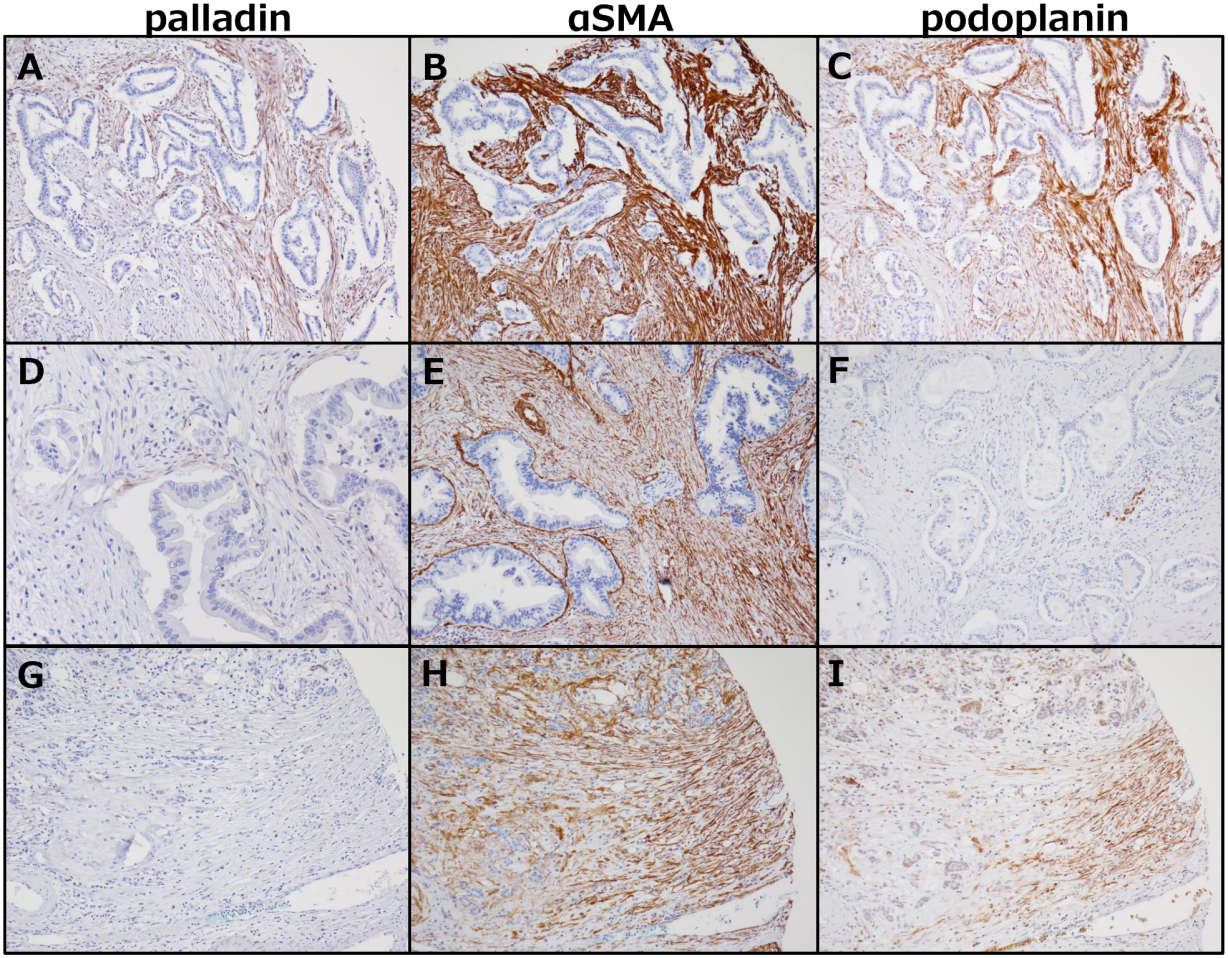
Immunohistochemical staining with palladin, podoplanin, and α-SMA antibodies. A-C were from the same core and stained with palladin (A, ×100), α-SMA (B, ×100), and podoplanin (C, ×100). These were regarded as positive. D: Few palladin-stained stromal cells are seen, but the proportion is <30% and categorized as negative (×200). E: Example of a podoplanin-negative case (×100). F: α-SMA-positive cells are seen diffusely, even in a case with relatively weak staining (×100). G-I: Pancreatic parenchyma outside the tumor is stained with each antibody, and podoplanin- or α-SMA-stained cells are seen abundantly, while this is not true for palladin (×100).

All TMA cases were examined for palladin, α-SMA, and podoplanin expressions in the stroma (Table 1), and representative pictures from different cases are shown in Fig 1. In terms of the staining patterns of palladin, α-SMA, and podoplanin, palladin-stained stromal cells and podoplanin-stained cells were mainly located near cancer cells, though podoplanin-stained cells were distributed to a wider area independent of cancer cell distribution. α-SMA was diffusely and nonspecifically stained regardless of the location and distance from the cancer cells. Stromal palladin expression was seen in 70 cases (41.9%), while podoplanin expression was observed in 131 cases (78.4%), and α-SMA expression was seen in all cases (100%). In pancreatic parenchyma outside the PDAC, palladin-positive cells were seen in only 8 cases (4.8%), though α-SMA-positive stromal cells were seen in 164 cases (98.2%), and podoplanin-positive cells were seen in 38 cases (22.8%).

**Table 1:** Immunostaining results for cancer-associated fibroblast markers.

Stromal expression	n (%)
Palladin	
Positive	70 (41.9%)
Negative	97 (58.1%)
α-SMA	
Positive	167 (100.0%)
Negative	0
Podoplanin	
Positive	131 (78.4%)
Negative	36 (21.6%)

α-SMA, α-smooth muscle actin.

### Correlations of CAF marker expression and other clinicopathological findings, including survival

**Fig 2:**
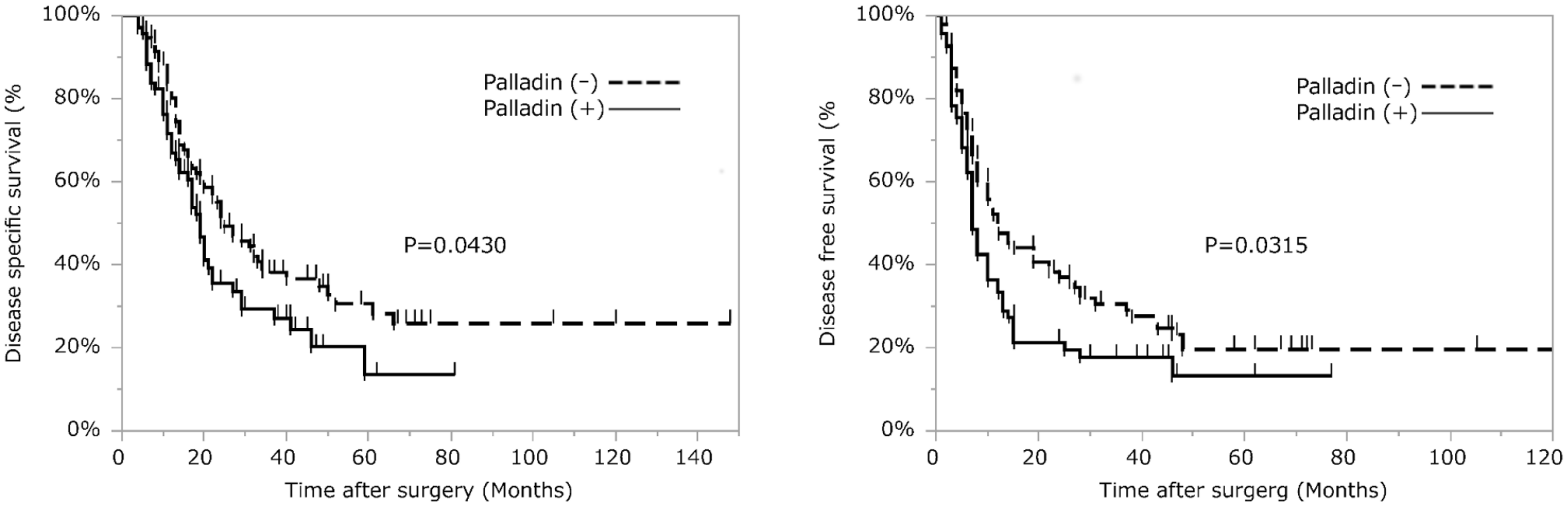
Survival analysis of pancreatic ductal carcinoma patients according to palladin expression. (A) Disease-specific survival and (B) disease-free survival. For disease-free survival, two cases were excluded due to lack of information about recurrence dates. Univariate analyses show that palladin expression is significantly correlated with unfavorable prognosis, both disease-specific survival and disease-free survival.

Palladin and podoplanin expression frequencies were positively correlated with each other (p = 0.0005), but no marker had a significant correlation with other clinicopathological findings (Table 2). The estimated five-year DSS rate was 23.1%, and the DFS rate was 16.8% in all patients. Univariate analyses showed that palladin expression was significantly correlated with unfavorable prognosis, both DSS (p = 0.0430) and DFS (p = 0.0315) (Fig 2). Other prognostic factors for DSS and DFS are listed in Table 3. Multivariable analysis using Cox regression models with the significant factors on univariate analysis was done for DSS and DFS, and palladin expression and some other factors were found to be independent prognostic markers for both DSS (HR, 1.60; p = 0.0243) and DFS (HR, 1.59; p = 0.0131) (Tables 4 and 5).

**Table 2:** Correlations between stromal palladin expression and other clinicopoathological factors.

	Stromal palladin expression	Positive (n = 70)	Negative (n = 97)	
		n (%)	n (%)	P-value^a^
Gender	Male	53 (61.4%)	55 (56.7%)	0.6331
	Female	27 (38.6%)	42 (43.3%)	
Age, y		66 (43–83)^b^	67 (48–89)^b^	0.8647^c^
Tumor location	Head	45 (64.3%)	53 (54.6%)	0.2651
	Body + tail	25 (35.7%)	44 (45.3%)	
Tumor size, cm		3.0 (1.2–8.0)^b^	3.0 (1.0–8.0)^b^	0.2630^c^
Histopathological grade	G1+2	59 (84.3%)	86 (88.7%)	0.4887
	G3	11 (15.7%)	11 (11.3%)	
Surgical margin	Positive	9 (12.9%)	15 (15.5%)	0.6631
pT	1+2	4 (5.7%)	3 (3.1%)	0.4543
	3+4	66 (94.3%)	94 (96.9%)	
Regional lymph node metastasis	Positive	52 (74.3%)	65 (67.0%)	0.3922
Distant metastasis	Positive	5 (7.1%)	6 (6.2%)	1.0000
Pathological stage	IA-IIA	16 (22.9%)	31 (32.0%)	0.2248
	IIB-IV	54 (77.1%)	66 (68.0%)	
Lymphatic invasion	Positive	48 (68.6%)	61 (62.9%)	0.5112
Vascular invasion	Positive	60 (85.7%)	78 (80.4%)	0.4139
Perineural invasion	Positive	64 (91.4%)	83 (85.6%)	0.3355
Recurent disease	Positive	56 (80.0%)	69 (72.6%)	0.4887
Local recurence	Positive	12 (17.1%)	17 (17.9%)	1.0000
Stromal α-SMA expression	Positive	70 (100.0%)	97 (100.0%)	N.A.
Stromal podoplanin expression	Positive	64 (91.4%)	67 (69.1%)	0.0005

α-SMA, α-smooth muscle actin; N.A., not available.

^a^P-values were calculated using Fisher’s exact test except values indicated by c, which were calculated using Wilcoxon’s test.

^b^The number in the box represents median (range)

**Table 3:** Prognostic factors for disease-specific survival and disease-free survival on univariate analysis.

	n	5-y DSS (%)	P-value^a^	5-y DFS (%)	P-value^a^
Gender					
Male	98	15.1	0.021	8.8	0.0552
Female	69	35.2		25.4	
Tumor location					
Head	98	20.7	0.0187	17.4	0.4171
Body + tail	69	30.6		17	
Tumor size					
≦3.0cm	94	33.2	<0.0001	26.5	<0.0001
>3.0cm	73	11.7		0	
Histopathological grade					
G1-2	145	27.5	0.0051	18	0.0004
G3	22	5.4		5.5	
Surgical margin					
Positive	24	23.9	0.0116	7.5	0.0125
Negative	143	25.3		18.2	
pT					
1+2	7	0	0.9999	0	
3+4	160	25.8		17	
Regional lymph node metastasis					
Positive	117	16.3	0.0149	8.2	0.0003
Negative	50	38.6		34.2	
Distant metastasis					
Positive	11	0	0.2408	0	0.5171
Negative	156	24.1		17.1	
Pathological stage					
IA-IIA	47	41.2	0.0029	36.4	<0.0001
IIB-IV	120	15.9		8	
Lymphatic invasion					
present	109	15	0.0006	9	<0.0001
absent	58	42.7		30	
Vascular invasion					
present	138	22.3	0.0509	13.9	0.0161
absent	29	36.6		31.9	
Perineural invasion					
present	147	20.4	0.0048	12.2	0.0018
absent	20	50.9		43.9	
Adjuvant chemotherapy					
yes	83	33.8	0.0084	18.1	0.1044
No	83	18.3		14.4	
Palladin expression					
Positive	70	13.5	0.043	13.2	0.0315
Negative	97	30.6		16.7	
Podoplanin expression					
Positive	131	18	0.175	0	0.1924
Negative	36	26.2		19.6	

DSS, disease-specific survival; DFS, disease-free survival.

^a^P-values were calculated using log-rank test.

**Table 4:** Independent prognostic factors for disease-specific survival on multivariable analysis.

	Hazard Ratio	P-value^a^	95% CI
Pancreas head tumor	1.65	0.0201	1.08–2.54
Tumor size (> 3.0 cm)	2.42	< .0001	1.58–3.69
pStageIIB—IV	5.79	0.0288	1.24–19.67
No adjuvant chemotherapy	1.59	0.0242	1.06–2.39
Palladin expression-positive	1.60	0.0243	1.06–2.41

CI, Confidence Interval.

^a^P-values were calculated using Cox regression models.

**Table 5:** Independent prognostic factors for disease-free survival on multivariable analysis.

	Hazard Ratio	P-value^a^	95% CI
Tumor size (> 3.0 cm)	2.30	< .0001	1.57–3.34
Histopathological grade G3	2.11	0.0126	1.18–3.54
Lymphatic invasion	1.78	0.0067	1.17–2.78
Palladin expression-positive	1.59	0.0131	1.10–2.28

^a^P-values were calculated using Cox regression models

### CAF marker expression in PDAC specimens after CRT

**Fig 3:**
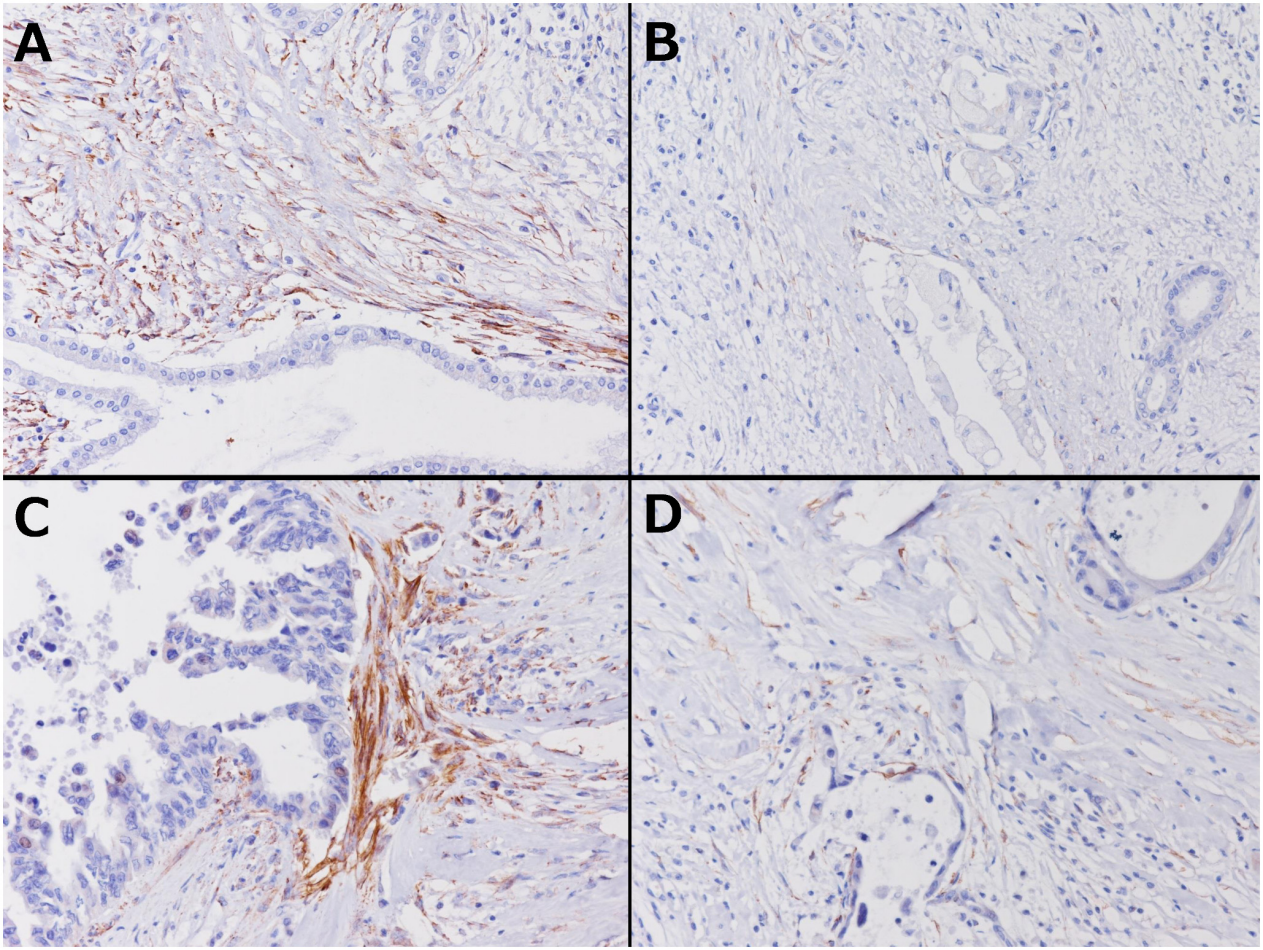
Immunohistochemical staining with palladin in treated cases. A and B are pictures of different areas from the same case treated before surgery, and C and D are from another treated case. (×200). Even in a case expressing palladin, the area and intensity of palladin staining are both decreased around degenerated cancer cells (B, D).

Second, whole sections from the SF and CF groups were examined immunohistochemically. There were no significant differences between the SF and CF groups in terms of the patients’ background characteristics. In the CF group, intravenous chemotherapy was administered to 16 patients, arterial infusion chemotherapy was added to 2 patients, and radiotherapy was added to 3 patients. One patient only took oral agents. The duration of preoperative treatment ranged from 2 to 44 months (median 7 months). The chemotherapeutic regimens consisted of simultaneous gemcitabine (GEM) and TS-1 in 10 patients, GEM and 5-fluorouracil in 3 patients, GEM alone in 6 patients, TS-1 alone in 1 patient, and GEM alone followed by TS-1 alone in 1 patient. The expression frequency of each CAF marker is summarized in Table 6. Palladin expression was seen in 16 cases (76.2%) in the SF group and 5 cases (26.3%) in the CF group, and the difference was significant (p = 0.0037). α-SMA expression was also seen more frequently in the SF group (p = 0.0177), but podoplanin expression was not (p = 0.3140). Palladin-stained stromal cell distribution was restricted to the area close to cancer cells; staining intensity was also weaker in the CF group, and palladin staining intensity around degenerated cancer cells was especially weak (Fig 3). Four in the SF group and one in the CF group (including one R1 case) had palladin-positive stromal cells on their surgical margin. Three of them had local recurrence, and exposure of palladin-positive stromal cells was correlated to local recurrence. (p = 0.0178)

**Table 6:** Stromal expression of cancer-associated fibroblast markers.

	SF group (n = 21)	CF group (n = 19)	
	n (%)	n (%)	P-value^a^
Palladin			
Positive	16 (76.2%)	5 (26.3%)	0.0037
Negative	5 (23.8%)	14 (73.7%)	
α-SMA			
Positive	21 (100.0%)	14 (73.7%)	0.0177
Negative	0	5 (26.3%)	
Podoplanin			
Positive	16 (76.2%)	11 (57.9%)	0.314
Negative	5 (23.8%)	8 (42.1%)	

SF, surgery first; CF, chemotherapy first.

^a^P-values were calculated using Fisher’s exact test.

**Fig 4:**
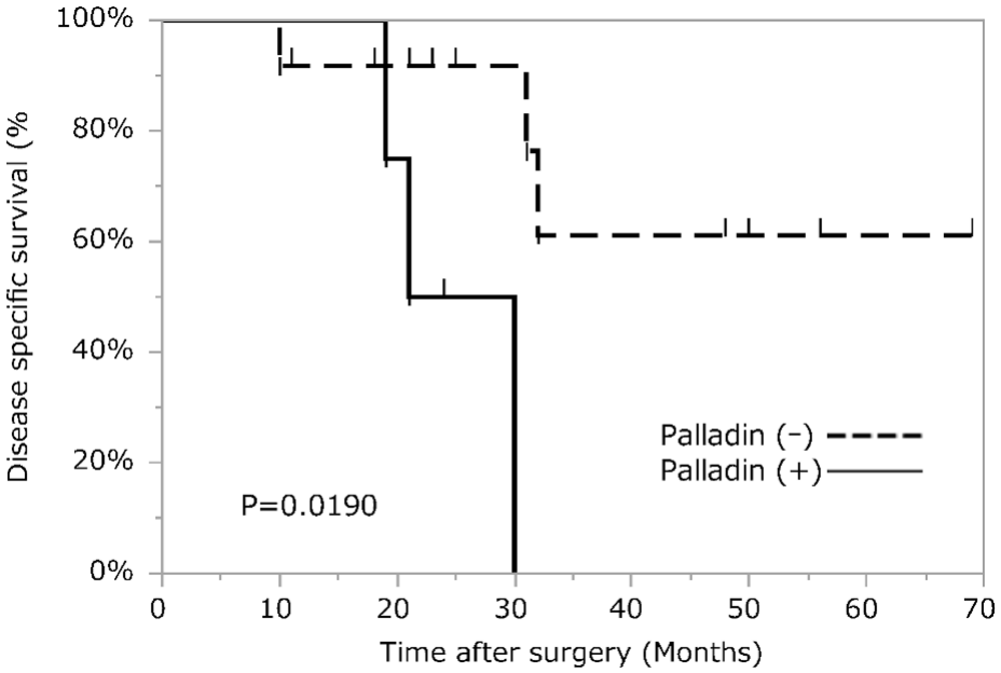
Disease-specific survival analysis of PDAC patients who underwent surgery after CRT based on palladin expression level. In a subanalysis of the chemotherapy-first group, palladin-positive cases have significantly shorter disease-specific survival after surgery.

The pathological therapeutic effects according to the Evans and CAP gradings in the CF group patients are shown in Tables 7 and 8 by palladin expression. Palladin expression had no correlation with treatment effects according to the grading systems. On survival analysis, there was a significant difference between the SF and CF groups in DSS; 5-year DSS was 28.7% in the SF group and 59.4% in the CF group (p = 0.0144). In the CF subgroup analysis, although the pathological therapeutic effect (Evans and CAP gradings) and α-SMA and podoplanin expressions were not correlated with DSS, palladin-positive cases had significantly shorter DSS after surgery (p = 0.0190) (Fig 4).

**Table 7:** Correlations of palladin expression and treatment-effect according to Evans grading.

		Stromal palladin expression		
Treatment-effect	Evans grading	Positive (n = 5)	Negative (n = 14)	P-value^a^
Low	I	2 (40%)	2 (14%)	0.106
	IIa	3 (60%)	5 (36%)	
High	IIb	0	4 (29%)	
	III	0	0	
	IV	0	3 (21%)	

^a^P-values were calculated using Fisher’s exact test.

**Table 8:** Correlations of palladin expression and treatment-effect according to CAP grading

		Stromal palladin expression		
Treatment-effect	The CAP grading	Positive (n = 5)	Negative (n = 14)	P-value^a^
Low	3	3 (60%)	2 (14%)	0.5304
	2	2 (40%)	8 (57%)	
High	1	0	1 (7%)	
	0	0	3 (22%)	

CAP, Colleague of American Pathologists.

^a^P-values were calculated using Fisher’s exact test.

## Discussion

The tumor microenvironment consists of various factors, including tumor cells, host immune cells, and stromal cells, that support or suppress each other. So far, the focus has been primarily on tumor cells rather than other factors, and this has contributed almost singularly to well-known prognostic factors related to the resected specimen, such as tumor staging, invasion, and remnant tumor. However, with the recent improvement in analysis of the tumor microenvironment, it has been found that not only tumor cells but also neighboring cells could affect patient prognosis. CAF is one of the stromal cells thought to contribute to poorer survival in various tumors including PDAC, which has been reported to contain high numbers of CAFs [10, 19–22]. Because of their ability to promote cancer cell proliferation, angiogenesis, invasion, metastasis, and immune regulation, CAFs are thought to be a potent therapeutic target, and many studies of CAFs have been conducted in PDAC [8, 9, 23].

Among them, various CAF markers have been suggested, including α-SMA, vimentin, fibroblast-specific protein-1, fibroblast activation protein, podoplanin, and palladin [15, 24–26], but they have some problems in specificity [27]. Of these markers, we investigated the relatively new CAF marker palladin. Palladin was originally reported as an actin-associated protein that appears to function as a potent cytoskeletal scaffold, and it is thought to be essential to embryonic cell motility and maintenance of cell morphology. A single palladin gene gives rise to multiple size variants (50 kDa, 90 kDa, 140 kDa, 200 kDa, etc.), some of which are expressed in tissue-specific patterns [28, 29]. While palladin-overexpression in pancreatic cancer is known [30], Salaria et al. suggested that palladin was dominantly expressed in stromal cells, rather than cancer cells, by immunohistochemical study and Western blotting [26]. However, there are partly inconsistent results regarding pallladin expression levels in PDAC cancer cells, compared with the present data. In the present study, palladin expression by PDAC cancer cells was scarcely seen as shown in Fig 1. This discrepancy in staining of cancer cells may be mainly because of the difference in the antibodies used in each study. The antibody they utilized reacts both 50 kDa and 90 kDa palladin proteins. However, in the present study, the same monoclonal antibody as Goicoechea et al. utilized to specifically detect the 90 kDa isoform among the palladin variants expressed in CAFs of PDAC was used [31]. Another possibility with respect to palladin expression in PDAC cancer cells is the presence of spindle-shaped tumor cells reported in other carcinomas, in which the cancer-related epithelial-to- mesenchymal transition (EMT) is developing [32]. Taking all this evidence into account, further in vitro study to clarify palladin specificity is warranted.

Furthermore, regarding for the function of palladin in the cancer microenvironment, Brentnall et al. reported that normal fibroblasts transfected with palladin gene were activated to become myofibroblasts, changing their morphology, and increasing the ability for cancer-cell invasion [13]. In addition, in mouse xenograft experiments, palladin expression levels in CAFs were significantly associated with tumor growth and metastasis of PDAC [33], while a correlation between palladin expression and an invasive phenotype has been demonstrated previously in a small set of patient samples. However, no previous studies have carefully tested the hypothesis that palladin levels can be used as a prognostic marker in pancreatic cancer. In the present study, a large scale analysis using the TMA method for CAFs of PDAC, in terms of palladin expression, was conducted for the first time.

Although palladin appeared to be specific as a CAF marker of PDAC, α-SMA and podoplanin did not show specificity to CAF, reacting with parenchyma both inside and outside tumorsindicationg its frequent expression in fibroblasts of non-tumoral tissue [34, 35]. Taking all these findings together, palladin was much more specific and better for use as an IHC marker of CAF, although both α-SMA and podoplanin also showed some dependency on tumor cells.

In this report, the clinical implications of palladin expression were also examined, and it was proven to be an independent prognostic factor in PDAC. Although palladin was reported to be a prognostic marker for renal cell carcinoma [15], to the best of our knowledge, no study has investigated the contribution of palladin to survival in PDAC, despite its specificity for CAF. This survival implication and previous reports of palladin promoting progression of PDAC in an experimental study may support each other [13, 33], but palladin expression was not correlated with other clinicopathological factors, including prognostic factors other than palladin, in the present study. This incompatibility could not be explained clearly due to the incompleteness of the investigation of the molecular mechanisms of palladin in PDAC; however, the incompatibility may partly be due to the fact that interactions of CAF and cancer cells are not completed by themselves alone, but also by others in the tumor microenvironment, including immune-associated cells such as macrophages and lymphocytes. Thus, palladin expression may represent tumor biology, which conventional prognostic factors cannot reflect. Taken together, palladin could be one of the potent CAF markers for assessing patients’ prognosis in PDAC. Moreover, stromal palladin expression in some other epithelial malignancies other than PDAC, such as lung, colon and gastric cancers, has recently been reported. [36]. Future research to examine the utility of palladin as a prognostic marker in these tumor types is warranted.

With the remarkable progress of preoperative CRT [4, 5, 37, 38], there have been many grading systems to evaluate therapeutic effects, starting with the Evans system, which have been mainly based on the proportion of residual viable or destroyed tumor cells, and some of these systems have also included aspects of fibrosis. [17, 18, 39]. However, many studies using these grading systems did not always show prognostic relevance [7, 40], partly because i) the word “viable” is subjective and has not been well defined, and ii) there is no guideline for distinguishing tumor-associated fibrosis, therapy-induced fibrosis, and background fibrosis [39]. With respect to this point, the present IHC results showed for the first time that palladin expression was significantly lower after CRT, and labeled stromal cells were scarcely seen, especially around the degenerated cancer cells. Despite this evident change, palladin expression did not correspond to post chemoradiotherapeutic histopathological grading, such as the Evans grading and the CAP grading; rather than these grading systems, palladin expression of itself was suggested to be a prognostic marker after CRT in this study. There are a few conflicting reports stating that CAF had increased after CRT in colorectal cancer and ovarian cancer, but IHC studies of PDAC after CRT were limited, and none used palladin as a CAF marker [41, 42]. Whether or how CAFs in PDAC changed after CRT was inconclusive, and this may depend on which marker was used. The present results suggest that decreasing CAF expression of palladin in IHC is another mechanism of the anti-cancer effect of CRT, which differs from destruction of cancer cells.

Another problem in assessing PDAC specimens was the existence of some locally recurrent cases despite R0 resection. In the present whole-section study, there were six such cases (four in the SF group, two in the CF group), and three (50.0%) of them had palladin-positive stromal cells to the surgical margin (p = 0.0178). This raises the possibility that CAF is a breeding ground for tumor recurrence in surgical margins. Part of the reason for local recurrence and palladin expression not being correlated in the TMA study may stem from heterogeneous distribution of palladin-expressing cells. It remains to be determined whether and how remnant CAFs play a role in local recurrence, and another study focusing on CAF in surgical margins appears needed. If it is elucidated, whether the fibrotic area where cancer cells disappeared after treatment should be included in the extent of resection may become clear.

In conclusion, stromal palladin expression was found to be an independent prognostic factor of PDAC, and it might also be a prognostic biomarker after CRT in PDAC. With further investigation of CAFs, it might be possible to establish more accurate post chemoradiotherapeutic grading systems and guidelines in PDAC.

## Supporting Information

S1 Table General characteristics of all Tissue Micro Array patients (n = 167). (XLSX)

S2 Table Background characteristics of patients in the second study using whole sections. (XLSX)

S3 Table Anonymized full data set of Tissue Micro Array study. (XLSX)

S4 Table Anonymized full data set of whole section study. (XLSX)
